# Supplementary material for: Assessing the Usability, Feasibility, and Engagement in IM FAB, a Functionality-Focused Micro-Intervention to Reduce Eating Disorder Risk
Source: Int J Environ Res Public Health. 2025 Oct 23;22(11):1618. doi: 10.3390/ijerph22111618 (PMC12652462; doi:10.3390/ijerph22111618)
Supplement: Supplementary file 1 [file ijerph-22-01618-s001.zip › ijerph-3859785-supplementary.pdf]

Supplementary Materials

Supplementary Table S1

Qualitative Responses to User Engagement Question Asking if they Would Engage in the IM FAB Program if Delivered via Smartphone App

**Comment Valence**	
Positive	
	I think that would be more helpful because then you could develop these good habits in your own home
	If this program was on an app I would use it all the time
	I would feel more comfortable.
	I do think it would be more convenient however I may not have actually paid as much attention if it was an at home activity
	It would definitely be more convenient.
	I think I would actually do it if it were on an app because I am also involved in psych studies and see why consistency is important. However, I think some students would not.
	I feel that I would use the app for a while then if i forget a day or two I would stop using it.
	The app would have been convenient
	I think its convenient and honors the privacy of individuals.
	That's a great idea! I would be open to it
	if i were to look at myself in the mirror and listen to all the good things my body does for me weekly, i would consider it a form of mediation and my eating, exercising, peace and body image would likely improve
	good idea!
	I do think that this raises self awareness and is a good way to think about what we actually care about in life.Sometimes we lose sight of whats's important or good in our lives due to the negatives that we focus on
	I like the gratitude prompts
	I liked looking at myself in the mirror for an extended period of time because I rarely do so.
	I really liked doing this study and I think my participation in it has been a great reminder and perspective giver. I would love to have reminders about all the things my body does for me every week and I'd definitely recommend it to others.
	It would make things a lot easier.
	The Smartphone app would be great for those who are judgmental of their body or going through a hard time.
	It would be smart. There may be more comfort for some people to have it on their personal device(s).
	I feel like it would be really beneficial to me, because it would help me appreciate myself and body more.
	I think it could be useful to a lot of people! Especially young girls! If I knew about the app, I would probably do a session or two, and then guide myself in

	the activity in the future.<br>I think that would be more helpful because then you could develop these good habits in your own home<br>If this program was on an app I would use it all the time<br>I would feel more comfortable.<br>I do think it would be more convenient however I may not have actually paid as much attention if it was an at home activity<br>It would definitely be more convenient.
Negative	
	I think I would definitely like this program better when I don't have to do it in between classes (when I am most stressed) and instead would enjoy it more as I am going to bed/just waking up.<br>I don't dislike my body, so I don't have need for mirror exposure.<br>If this program was through an app, there is a chance that If I were to use it, it would start to become detrimental and cause negative effects, which is most likely not the intention. I would become more attentive to each part of myself and may become more critical which would not be good for myself or for those around me. While there are some times where looking in a mirror at specific parts may help, if the current week was not good, or if I already didn't think too highly about myself, it may just further exacerbate the issue.<br>For me I don't have a strong intension to use a smartphone app like this, because this do make me feel that someone if persuading me that the appearance is not as important as the functions. I don't like the feeling of pushing me toward an idea and I don't acutely feel it can provide me a positive gratitude.<br>Using this program in an app would be easier and it might cause more people to do it. Since it is out of my own free-will that I am doing this, I do not see why I would not have done some of the activities if I was at home.<br>While at school, I would be unwilling to participate from my own room because of my roommates being there<br>The app would be solely dependent on individuals willingness to integrate the app in their everyday lives. I think there are many girls and boys I interact with on a daily basis that are probably on the disordered eating spectrum but are not diagnosed and treated accordingly because it is so common (especially at this school) where people do not recognize it as an issue. Which would ultimately limit the success of the app if girls and boys are not genuinely educated on it. Though moreover, individuals who already have eating disorders or disordered eating often would disregard the benefits this kind of app would provide because they don't truly want to stop. But if individuals do want to stop and control their eating disorder or disordered eating perhaps this app would be beneficial because the road to full recovery is a long process where some people may not have the family support system that others have but an app like this could help them along.<br>I know people who I would recommend the app to, but I think the mirror exposure might not be great for my own self image, so I'm not sure I would just do this on my own.<br>I believe that a mood tracking app would help, but the mirror portion I do not believe that people would take that seriously at home.

Neutral	
	If it wasn't too time-consuming I'd do it.
	I would not care. Either way is fine with me.
	it's hard to arrange a mirror to frame your entire body
	I think it would be a cool app i just dont know if i would actually use it
	I would recommend to indicate to the person to be alone during the sessions to feel the most conformable and if the app can track the days and moods of the person from day to day.
	I may not actually follow through. I enjoyed coming to a site.
	Doing it via app makes it so that you would have to set aside time to do it for yourself. I do not think that I would unless I was feeling extremely insecure about my body.
	I just could not see myself being accountable with an app. If I wanted it for my own purposes maybe, but for research purposes, I would've just turned it on and walked away.
	it would be easier than having to come in to the lab
	I feel that I could use an App like this now, but without this experience I likely wouldn't take it seriously enough to see any improvement by myself.
	I would be interested in using an app similar to the study but it depends on what the app did. If it was just instructing me to observe my body I probably wouldn't use it because I already do that on a regular basis. If it provided other insights like how to appreciate my body more and other health facts, I would probably use it.
	I am not sure because I would have more distractions on my phone whereas on my laptop I devote my full attention.
	The video is a tad too long speeding it up would make it more effective on the fast pace world we live in. No one wants to sit and listen for longer than 5-8 minutes.
	It would be more accessible but I think it is still capable on a laptop
	It'd be handy, but could be confusing

Measures:

UMUX = usability of current system
We are interested in understanding how "usable" the study was for you. In other words, how easy or hard it was to do the things that were asked practically (rather than emotionally)? Were there any frustrating aspects of the study set-up that you would change or fix were we to run a similar study in the future? Please answer the next few questions and provide us with any additional details or thoughts you have in the open-ended response section.

Rating Scale: 1 = strongly disagree - 7 = strongly agree

1. The study's procedures and capabilities (mirror sessions & text prompts) meet my requirements.
2. The study's procedures (mirror sessions & text prompts) are a frustrating experience.
3. The study's procedures (mirror sessions & text prompts) are easy to use.
4. I have to spend too much time correcting things with this study's procedures (mirror sessions & text prompts).

Please provide use with any additional feedback on the ease of use of the study's procedures: [open-ended response]

Feasibility of app-based delivery
We are interested in understanding your opinions on doing a similar program at home using an Smartphone App that prompted you to do the weekly mirror exposures and regularly texted you to prompt you to practice gratitude, with a few short questionnaires being asked directly on the phone before and after the mirror exposure sessions. Please answer the next few questions and provide us with any additional details or thoughts you have in the open-ended response section.

Rating Scale: 1 = strongly disagree - 7 = strongly agree

1. I would have preferred doing this program if it were fully app-delivered.
2. I would have preferred doing the mirror exposure at home if it was on an app.
3. I would probably not have actually done the mirror exposures at home if the study was delivered via an app.
4. I would use an app like this in my own life.
5. I would recommend a program like this on an app to a friend or relative.

Please provide use with any additional feedback on how you would feel about doing a similar program on your own via a Smartphone App: [open-ended response]

Engagement with program
We are interested in understanding how you felt about the study procedures (emotionally, rather than procedurally or practical issues). Please answer the next few questions and provide us with any additional details or thoughts you have in the open-ended response section.

Rating Scale: 1 = strongly disagree - 7 = strongly agree

1. I found the mirror exposure engaging or interesting.
2. I found the texting assignments engaging or interesting.
3. I thought that the mirror exposure was uncomfortable and distressing.
4. I felt that the mirror exposure was helpful.
5. I thought that the gratitude texts were uncomfortable and distressing.
6. I felt that the gratitude texts were helpful.

7. I would recommend this study (or doing the same mirror exposure and gratitude texting) to a friend or relative.
8. I was bored during the mirror exposure sessions.
9. I was bored when responding to the gratitude texts.

Please provide use with any additional feedback on your opinions about the mirror exposure and gratitude texts. Did you notice any changes during the study? Did you feel that they were helpful, distressing, harmful, or did not impact you much in one direction or the other? We are interested in any and all feedback you have about this self-image program. [open-ended response]

Manipulation Check Questions

1. As part of this research study, were you asked to imagine yourself as a kangaroo living in the outback. Y/N
2. As part of this research study, were you given lists of vocabulary words to memorize? Y/N
3. As part of this research study, were you asked to take a math test? Y/N
4. As part of this research study, have you been asked to listen to an audio recording? Y/N
5. As part of this research study, were you asked to look in a mirror? → logic to skip if N

These questions pertain directly to the audio recording that you just listened to and the thoughts, behaviors, and feelings you experienced as you were looking in the mirror.

6. When you were looking in the mirror, were you asked to examine specific body parts? Y/N
7. When you were looking in the mirror, were you asked to think about what those body parts enabled you to do or experience? Y/N
8. When you were looking in the mirror, were you asked to think about what you were grateful for regarding your body? Y/N

Please rate the following based on how they fit with your experience while you were looking in the mirror a few minutes ago (10 item, visual analogue scale, from *not at all* to *extremely*)

9. When I was looking in the mirror, I felt anxious.
10. When I was looking in the mirror, I felt disgust.
11. When I was looking in the mirror, I appreciated my body.
12. When I was looking in the mirror, I had positive thoughts about my body.
13. When I was looking in the mirror, I had negative or critical thoughts about my body.
14. When I was looking in the mirror, I accepted my body as it is.
